# Patients’ views and experiences of delayed diagnosis of inflammatory bowel disease: a qualitative study

**DOI:** 10.3399/BJGPO.2023.0070

**Published:** 2023-11-29

**Authors:** Eleanor Cross, James A Prior, Adam D Farmer, Benjamin Saunders

**Affiliations:** 1 School of Medicine, Keele University, Keele, UK; 2 Midlands Partnership University NHS Foundation Trust, Trust Headquarters, St. George’s Hospital, Stafford, UK; 3 Department of Gastroenterology, University Hospital of North Midlands NHS Trust, Stoke-on-Trent, UK

**Keywords:** embarrassment, delayed diagnosis, diagnosis, general practice, inflammatory bowel diseases, primary care, qualitative research, uncertainty

## Abstract

**Background:**

Diagnosing inflammatory bowel disease (IBD) can be challenging. Patients have been found to experience significant diagnostic delay, which can lead to poorer clinical outcomes. The reasons for this delay are not fully understood, and exploring patients’ perspectives can offer important insights.

**Aim:**

To explore the views and experiences of patients who self-report a delay in IBD diagnosis.

**Design & setting:**

Qualitative methodology using semi-structured interviews. Participants were recruited via social media and a national IBD charity.

**Method:**

Interviews were conducted by telephone between December 2018 and February 2019. Data were analysed using thematic analysis and drawing on the constant comparison method.

**Results:**

Sixteen interviews were carried out. Ten participants were female and six were male; participants were aged 20–65 years. Four main themes were identified: patient factors contributing to delay; primary care factors contributing to delay; systemic factors contributing to delay; and perceived consequences of delayed diagnosis. Participants reported initially not seeking help due to embarrassment or normalising their symptoms. Having consulted, participants reported further delay in receiving a diagnosis due to their perception that GPs had either mislabelled symptoms, expressed uncertainty, or not taken symptoms seriously. Systemic factors, including lack of access to test results and communication issues across primary and secondary care, were also cited as contributing to delayed diagnosis. Several participants felt that their delayed diagnosis led to poorer clinical outcomes.

**Conclusion:**

These findings can support patients and GPs in their conversations about symptoms that may indicate IBD, and potentially contribute to reducing diagnostic delay, as well as informing future primary care interventions.

## How this fits in

Patients with inflammatory bowel disease can experience significant diagnostic delay, but reasons for this delay are not fully understood. Through exploring patients’ views and experiences, several barriers to diagnosis were identified. These included patient, primary care, and systemic factors. These findings can support patients and GPs in their conversations about symptoms, and potentially contribute to reducing diagnostic delay in IBD.

## Introduction

Inflammatory bowel disease (IBD) is an umbrella term for a group of inflammatory conditions of the colon and small intestine, of which the major types are Crohn’s disease (CD) and ulcerative colitis (UC). Both conditions present with similar symptoms, although weight loss, diarrhoea, and fatigue are most common in CD, and rectal bleeding and urgency to defecate are more often associated with UC. In the UK, it is estimated that 600 000 people have IBD.^1^ IBD management centres around immunosuppressive medications, although surgical intervention may be indicated in severe disease.^2^

The diagnosis of IBD is commonly made through internal investigation in the form of a colonoscopy or sigmoidoscopy. Since 2013, faecal calprotectin has been available in the NHS, a screening test to differentiate between inflammatory and non-inflammatory causes of symptoms.^3^ If screening via faecal calprotectin or blood test indicates a need for further investigation, a colonoscopy or sigmoidoscopy is carried out to visualise the bowel and take biopsies, and to confirm an IBD diagnosis. The importance of early identification and diagnosis of IBD is highlighted in National Institute for Health and Care Excellence (NICE) guidelines,^4^ which assert that *‘a delay in assessment and diagnosis can be associated with adverse consequences, such as clinical complications and a negative effect on the person’s quality of life’*. This is supported by recent research indicating that delays in diagnosis of CD and UC are associated with an increased risk of intestinal surgery.^5^ However, evidence suggests that diagnostic delay in IBD is common; a recent systematic review showed median diagnostic delay for adults ranges from 2–26 months for CD and 2–12 months for UC.^6^ The review included studies conducted in Europe (two from the UK), Asia, North America, and South America, with the majority (80%) published after 2006.

The reasons for delay in IBD diagnosis are not yet fully understood, but one explanation could be the non-specific nature of symptoms, which can be mistaken for other more common conditions like irritable bowel syndrome (IBS) or haemorrhoids.^7^ The reasons for the lengthier delays in CD compared with UC could be because the location of disease in UC is confined to the large bowel, and patients experiencing rectal bleeding due to UC may be more likely to present to their doctor. Patients with CD may present with a wider range of non-specific symptoms, such as abdominal pain or constipation, which may be more commonly associated with other conditions with overlapping symptoms.^6^

To better address the extent and impact of delayed diagnosis of IBD, we need to understand the barriers to identification and diagnosis, and to find ways to support clinicians and patients to overcome these barriers. In turn, this can have important implications for improving clinical outcomes and reducing associated healthcare costs. Early identification of IBD is of particular relevance in primary care, as most patients initially present to their GP with symptoms. A recent UK cohort study identified 142 cases of IBD per 10 000 patients consulting in UK primary care, a significant increase on figures from previous decades.^8^ To date, however, no research has investigated the reasons for diagnostic delay across primary care and secondary care, and there have been no qualitative studies exploring the perspectives of patients with IBD on diagnostic delay. This study aimed to explore the views and experiences of patients who self-reported experiencing a delay in IBD diagnosis.

## Method

One-to-one semi-structured interviews were carried out via telephone between December 2018 and February 2019 with individuals who self-reported a diagnosis of IBD and had experienced a delay in receiving their diagnosis. This article conforms to appropriate qualitative reporting guidelines.^9^

### Eligibility and recruitment

Individuals aged ≥18 years with a self-reported diagnosis of either CD or UC and a delay in receiving their IBD diagnosis of at least three months were recruited for interview. It was specified that participants must have received, or be currently receiving, care through the NHS. Individuals with a diagnosis of indeterminate colitis were excluded. A convenience sampling approach was taken, with participants self-selecting based on meeting these criteria.

Participants were recruited through social media advertisements on Twitter and Facebook, and on the website of the IBD charity Crohn’s and Colitis UK. The study was also advertised at a regional Crohn’s and Colitis UK event. Individuals interested in participating first contacted the research team either by telephone or email, following which they were sent a formal invitation letter, participant information sheet, and consent form by email. Completed consent forms were returned by post or email, following which interview arrangements were made.

### Data collection

Telephone interviews were conducted by the lead author (EC), a medical student with training in qualitative methods. Interviews aimed to gain in-depth understanding of participants’ experiences of receiving an IBD diagnosis and their views on the reasons for their diagnosis being delayed. Interviews were between 30 minutes and 1 hour in length. A topic guide was used to inform interviews (see Supplementary Information S1); however, the interviewer retained flexibility to follow up on any unexpected findings emerging during interviews. The topic guide was developed based on the study aims and previous literature in the IBD field, and was iteratively revised throughout the data collection process based on emergent findings. For instance, in early interviews participants explicitly talked about their emotional reaction to delayed diagnosis, which was added to the topic guide and further explored in subsequent interviews. Data collection continued until sufficient data saturation was judged to have been achieved. Saturation was assessed at the level of the initial coding of the data, whereby sufficient saturation was felt to have been achieved when new information was no longer identified from the data; this was referred to as ‘informational redundancy’.^10^ It was at this point that data collection ceased. All interviews were audiorecorded, and informed consent was reaffirmed at the beginning of interviews.

### Data analysis

Audiorecordings of interviews were fully transcribed and anonymised. Data were analysed using thematic analysis, following the stages outlined by Braun and Clarke,^11^ and also drawing on the constant comparison method,^12^ to highlight data consistencies and variations. To identify recurrent concepts inductively, anonymised transcripts were first systematically coded on a line-by-line basis by EC, with the aid of the software programme NVivo (version 12).

Codes with similar meaning were then grouped into higher-order codes, and mind mapping was used to explore connections between these codes. A series of meetings between the cross-disciplinary team was then held, which involved moving back and forth between looking at the higher-level codes created and the data, and beginning to discuss interpretations of these codes and data beyond the purely descriptive level. These team discussions led to the identification of themes and subthemes that reflected patterns observed across the dataset.

## Results

### Participant characteristics

In total, 16 participants aged between 20 and 65 (mean age = 41) years were interviewed. Ten participants identified as female and six as male. Four participants were diagnosed with UC and 12 with CD. The mean age of participants at diagnosis of IBD was 29 years, ranging from 15–60 years. Six of the seven participants who were currently in paid employment worked in professional or office-based roles. Of the rest, three were university students, four were retired, and two were unemployed. The median diagnostic delay from symptom onset to diagnosis was 6 years (Table 1).

**Table 1.** Participant characteristics

Gender	IBD phenotype	Occupation	Age symptoms began, years	Age at diagnosis, years	Diagnostic delay, years	Current age, years
Female	UC	Health care	40	40	<1	53
Female	UC	Health care	16	30	14	34
Male	UC	Professor	8	10	2	62
Female	UC	Student	12	16	4	18
Female	UC and CD	Student	14	15	1	28
Male	CD	Student	9	17	8	25
Female	CD	Unemployed	16	23	17	38
Female	CD	Retired	50	60	10	65
Male	CD	Retired	Early childhood	50	50	57
Female	CD	Teacher	25	26	1	45
Female	CD	Office work	16	19	3	22
Female	CD	Office work	10	18	8	20
Male	CD	Patient transport	19	20	1	23
Female	CD	Unemployed	16	42	26	45
Male	CD	Retired	36	47	11	65
Male	CD	Retired	29	31	2	56

CD = Crohn’s disease. IBD = inflammatory bowel disease. UC = ulcerative colitis.

### Main themes

Four main themes, with related subthemes, were identified:

1. Patient factors contributing to delay

- Subthemes: embarrassment contributing to delayed help-seeking; normalising symptoms; and fear of symptoms indicating severe illness

2. Primary care factors contributing to delay

- Subthemes: initial mislabelling of symptoms; perceptions towards GP management of symptoms; and referral to different secondary care services

3. Systemic factors contributing to delay

- Subthemes: administrative factors; delays in receiving secondary care appointment; and communication between primary and secondary care professionals

4. Perceived consequences of delayed diagnosis

- Subthemes: perceived impact of delay on symptoms and subsequent surgery; impact of delayed diagnosis on mental health; and delayed diagnosis not perceived as negatively impacting condition

#### Theme 1: Patient factors contributing to delay

Several participants reflected on having delayed initial help-seeking for their symptoms for several weeks or months, which they felt had contributed to their delay in receiving a diagnosis of IBD.

##### Embarrassment contributing to delayed help-seeking

Reasons reported for delayed help-seeking included embarrassment in consulting the GP with symptoms such as diarrhoea and bloody stools, which may reflect a taboo related to discussing bowel-related issues:

*‘I think embarrassment is a terrible sort of thing, but you do put it off as long as you can … you give yourself all sorts of excuses.’* (Female [F], aged 65 years, CD, 10-year delay)

##### Normalising symptoms

Others reported ignoring or normalising their symptoms through attributing symptoms to life stressors as a reason for not initially consulting their GP:

*‘I did put it down to just like stress at work, you know,* [inspection at work] *and stuff like that, I assumed because I’d never been ill, I’d hardly ever been ill before … I thought I’d just go back to how I was and I think maybe there is a bit of burying your head in the sand sometimes as well, you know, maybe knowing things aren’t quite right but hoping they’d just sort themselves out on their own.’* (F, aged 45 years, CD, 1-year delay)

##### Fear of symptoms indicating serious illness

Alternatively, some participants were afraid of their symptoms potentially indicating a serious illness that would lead to them being hospitalised, underlying their reluctance to consult the GP:

*‘I think I was morbidly afraid of being taken into hospital because I’d never been in hospital or anything and I had a real thing about not going into hospital, so I didn’t want to particularly, maybe face it*.*‘* (Male [M], aged 65 years, CD, 11-year delay)

Participants reported that a worsening of their symptoms, to the point that they did not feel help-seeking could be avoided, eventually led them to consult the GP:

*‘I was just getting worse, you know, and it got to the stage where I couldn’t leave the house and I tried to go back to work in the September but I think I barely did a week and it was obvious that wasn’t going to work so yeah I think at that stage I was going* [to the GP]*.’* (F, aged 53 years, UC,<1-year delay)

### Theme 2: Primary care factors contributing to delay

#### Initial mislabelling of symptoms

Participants reported that when they did seek help for their symptoms, they faced further delays. Many reported that their symptoms were initially mislabelled, for example, gastroenteritis, and later IBS. Some felt that their symptoms had not been sufficiently investigated before potential diagnoses were suggested:

*‘I went to my GP … and they just said, “ooh you’ve got a tummy bug”, first of all and when it didn’t clear up, “it’s probably IBS”. You know, slightly different things but not sending me for any exploratory tests or anything. So just sort of saying, “it’s probably this, it’s probably that” and not really giving me any answers.’* (F, aged 38 years, CD, 17-year delay)

#### Perceptions towards GP management of symptoms

When symptoms did not improve through IBS management, participants felt GPs persevered with ineffective management rather than considering alternative explanations:

*‘It just seemed to be forever pepperminty things, you know drinks and tablets … I don’t recall anything else coming to light, no … just kept treating it as IBS, which was a bit frustrating*.*‘* (F, aged 28 years, CD, 1-year delay)

Several participants also reported feeling that their GP initially did not take their symptoms seriously, and as a result delayed referring them for further investigations:

*‘Probably once a week, I just used to go down to the doctor and go, “Can you help me, can you refer me?”, and they just used to fob me off, peppermint capsules* et cetera *and I just went every single week for what must have been eight or nine weeks, that’s when someone actually referred me* [to a gastroenterologist] *so yeah, I must have been to the GP 30 times*.*‘* (F, aged 22 years, CD, 3-year delay)

Others reported their GP expressing uncertainty about the cause of their symptoms:

*‘It’s more that the doctor couldn’t work out what was happening. So, he didn’t see it so, I remember the conversations, well some of them, I remember the conversation emphasis was, “I don’t know what’s happening”*.*‘* (M, aged 62 years, UC, 2-year delay)

There was one example, however, which appeared to be a deviant case within our dataset, in which the participant reported a GP identifying their symptoms as potentially indicating IBD in the initial consultation. The participant reported that they had already seen several different GPs when presenting with the same symptoms before this consultation:

*‘She looked through my medical history and she took time; I think that was the difference. She knew I was unwell; she did my observations; they weren’t obviously good because I wasn’t very well. But she took time to read about my past medical history, she’s an out-of-hours doctor, but she’s a fresh pair of eyes, and then that’s when she said, “Has anyone ever told you, you could have colitis?”’* (F, aged 34 years, UC, 14-year delay)

#### Referral to different secondary care services

A few participants reported that they were initially referred to different secondary care services by their GP before later referral to a gastroenterologist who diagnosed IBD. These participants felt that this had contributed to the delay in receiving a diagnosis:

*‘I got the referral for gastro, but I’d been first to other places like colorectal surgeons and everything … I think it was about three times* [receiving a different referral]*. I saw two colorectal surgeons and then I think I saw, what’s the one that deals with hormones and things, gynaecologist* [sic] *endocrinologist.’* (F, aged 22 years, CD, 3-year delay)

### Theme 3: Systemic factors contributing to delay

#### Administrative factors

Participants described organisational and administrative factors that they felt contributed to their delayed diagnosis. This included difficulty in accessing test results:

*‘They did do a calprotectin. I never heard back and I was told, “If you don’t hear back, everything is fine”. Six months later, I lost loads of blood. My mum said, “That’s absolutely definitely not fine. That’s not normal.” I rang up and I said, “What were my calprotectin results six months ago?” They refused to give it me. I still haven’t had that and I’ve been told now that it’s been lost.’* (F, aged 20 years, CD, 8-year delay)

#### Delays in receiving secondary care appointment

Having been referred to a consultant by their GP, some participants reported experiencing long waiting times to receive an appointment:

*‘The blood was getting more and more like every day, my mum took me back to the GP and then he said, we need to pursue it a bit further and investigate it, and then sent me for an urgent referral, but even that took about five to six weeks to be urgently referred*.*‘* (F, aged 28 years, CD, 1-year delay)

#### Communication between primary and secondary care professionals

For some participants, even once they had received a confirmed diagnosis of IBD following a delay, there were then further delays in beginning treatment due to communication issues between consultants and GPs about medication to be prescribed:

*‘When I had my appointment to go back to see the consultant ... he said, “How are you getting on with your Asacol?”, and I said “What’s Asacol?”, and he said, “It’s what I told your GP to give you when we diagnosed your colitis”. I said “Well I’ve not had any”; so he was a bit cross about that and told me to go back to the doctor and find out why I hadn’t been given it and it was just that because I was well, I wasn’t moaning or nagging they just didn’t prescribe it*.*‘* (F, aged 53 years, UC,<1-year delay)

### Theme 4: Perceived consequences of delayed diagnosis

#### Perceived impact of delay on symptoms and subsequent surgery

Participants reported consequences they felt had resulted from delays in diagnosis. Many participants felt that delays had led to a worsening of their symptoms, and subsequently their IBD had been more difficult to manage:

*‘It took me over a year of being on infliximab to actually not having any symptoms anymore. I had to try numerous other medications before that. It took over 18 months to actually get to the point where I hadn’t got any symptoms anymore … delayed diagnosis has an impact because it’s more severe and so I’ve found that it’s harder to treat*.*‘* (F, aged 20 years, CD, 8-year delay)

Others felt that an earlier diagnosis may have prevented the need for surgery:

*‘I wonder if maybe I wouldn’t have gotten a stricture and because when I had my stricture removed, when I had my operation, they said there was an awful lot of adhesions … and it was mentioned to me that it could have been because I’d had Crohn’s symptoms for such a long time ... and I wonder if I may not have had a stricture if I’d gotten all the right medication earlier*.’ (M, aged 23 years, CD, 1-year delay)

#### Impact of delayed diagnosis on mental health

Some participants reported that delayed diagnosis had an impact on their mental health and that of their family:

*‘When you feel ill every single day it’s really draining and when someone is not listening to you and just keeps sending you away it’s really very frustrating, I think my mum was just getting more and more worried because I’d just lose more weight, “you look worse, you are anaemic”, it wasn’t good, it wasn’t a good time.’* (F, aged 22 years, CD, 3-year delay)

#### Delayed diagnosis not perceived as negatively impacting condition

There was some variation in the data, as a few participants did not feel that delayed diagnosis had negatively impacted the course of their condition:

*‘I think in the long term the delayed diagnosis hasn’t had* [an impact]*, because I did get back on my feet, and obviously since then it’s been the path of any other chronic disease, I’ve had periods when I’m fine and periods where I’m really not fine and I don’t think that would be any different if I’d been diagnosed quicker.’* (F, aged 45 years, CD, 1-year delay)

## Discussion

### Summary

To the authors’ knowledge, this study is the first to explore the views of patients with IBD on the reasons for their delayed diagnosis, and perceived consequences of this delay on their condition. Several reported barriers to diagnosis were identified related to the patients’ help seeking, identification and management in general practice and secondary care, and systemic factors. Most participants felt that diagnostic delay led to a worsening of their symptoms, negative impacts on wellbeing, and/or need for surgery.

### Strengths and limitations

The exploratory approach adopted in this study allowed for the identification of a range of different factors related to diagnostic delay of IBD. The multidisciplinary team involved in data analysis was also a strength, which increases the trustworthiness of the findings presented. However, the team did not include anyone currently working in primary care, which would have added a useful perspective to the analysis.

A limitation of the study is that individuals who reported having experienced delayed diagnosis were purposefully recruited. As such, more severe, ‘interested’ cases may have been recruited to the detriment of those with more positive experiences of receiving a timely diagnosis. However, the authors wanted to focus on barriers to delay and, as will be explained further below, some findings do align with the most recent IBD patient survey,^13^ suggesting they may have broader relevance irrespective of these sampling choices.

Additionally, some participants had been diagnosed with IBD a number of years previously and there may have been issues with recalling events. Their experiences of diagnosis did not reflect the contemporary healthcare context, including more recent advances in testing such as the availability of faecal calprotectin. However, there were similarities between the experiences of those participants diagnosed several years ago and those diagnosed more recently.

Another limitation is that information on participants’ ethnicity, cultural background, or socioeconomic status were not collected, limiting the ability to compare experiences of diagnostic delay based on these characteristics. However, job status and occupation were reported, which may be a proxy for socioeconomic status.

When interpreting the findings, it is important to acknowledge the influence of the researcher on participants’ interview responses. However, a reflexive approach was taken, with the interviewer attending to and acknowledging any biases and preconceptions. Participants were made aware that the interviewer (EC) was a medical student. This could have led participants to feel hesitant about expressing negative views about their health care. However, participants did disclose healthcare barriers to timely diagnosis, suggesting this did not lead to hesitancy.

### Comparison with existing literature

These findings align with research that has identified negative consequences of delay for disease course. Several participants in the present study perceived that their delayed diagnosis resulted in poorer disease outcomes. A recent systematic review and meta-analysis identified that delayed diagnosis in CD was associated with a higher probability of stricturing (that is, narrowing of the intestines), penetrating disease, and intestinal surgery. In UC, delayed diagnosis was associated with higher odds of colectomy (surgical removal of the colon).^5^

The present study findings can be compared with those from the most recent UK IBD patient survey,^13^ completed by 10 222 patients with IBD. While average time to diagnosis was not reported, more than one-quarter of survey responders (26%) reported waiting >1 year for a diagnosis. Some of the views on reasons for diagnostic delay show similarities with the present study findings, such as symptoms being mislabelled as IBS. However, other factors identified, such as patients’ reports of having received referrals to different secondary care services before later being referred to a gastroenterologist, were not reported in this survey.

Outside of the IBD literature, comparison can be drawn with qualitative research on the diagnosis of other inflammatory and bowel-related conditions. A study on the views of people living in New Zealand with colorectal cancer on the barriers and facilitators to diagnosis similarly identified participants normalising bowel symptoms, and therefore delaying initial help-seeking.^14^ Their participants also reported mislabelling of symptoms as being caused by less serious illnesses such as IBS. A forthcoming study on the barriers to diagnosis of axial spondyloarthritis (axSpA, a type of autoinflammatory arthritis), which explored the views of people with axSpA and health professionals, also made some similar findings (Hay *et al*, unpublished data). For instance, the difficulty in differentiating between axSpA and other conditions with similar symptoms, such as osteoarthritis and fibromyalgia, was highlighted as a key barrier. This indicates that some of the barriers to timely diagnosis identified in the present study may also be common to other long-term conditions.

### Implications for research and practice

The present study findings have implications for enabling earlier identification and diagnosis of IBD. Hesitancy from patients to initially seek help due to lack of awareness of IBD and embarrassment about symptoms was identified. Raising awareness of the symptoms of IBD through public health messaging, and campaigns tackling the taboo around consulting health care with bowel-related symptoms, could encourage earlier primary care help-seeking. Awareness campaigns have been found to be effective for bowel cancer, which has some similar symptoms to IBD.^15^

Patients reported that they felt that their GP was uncertain about the cause of their symptoms. Education and training for primary care professionals in the signs and symptoms of IBD, particularly in terms of differentiating IBD from conditions with overlapping symptoms such as IBS, could enable earlier identification in primary care. This may include encouraging GPs to engage with existing training resources. For instance, the Royal College of General Practitioners have recently collaborated with Crohn’s and Colitis UK to produce an IBD toolkit to educate GPs about the identification and management of IBD.^16^ This toolkit, in line with current NICE guidance for diagnosis of UC and CD,^17^ advises clinicians to consider these diagnoses in the presence of symptoms such as unexplained persistent diarrhoea for >4–6 weeks, and if available, to consider arranging screening in primary care, such as serum full blood count or faecal calprotectin. The present study findings align with this guidance; however, in current guidance and training there is an absence of patients’ perspectives towards delay, as presented in this study, which can be useful in informing patient–clinician conversations. For instance, several patients communicated the concern that they did not feel that their symptoms had been taken seriously by their GP. While it is important to highlight that this only represents patients’ perspectives, these findings suggest that greater exploration and discussion of patients’ concerns about symptoms within consultations may be useful, as well as taking a shared decision-making approach to management and referral decisions. This could be of benefit clinically in helping GPs to consider other causes of symptoms, aside from more common explanations like IBS. It could also benefit therapeutic rapport and provide reassurance to patients that their concerns have been taken into account, all of which could positively affect patients’ outcomes. It would be useful for future research to explore the views of primary care practitioners towards diagnostic delay for IBD to add further evidence to patients’ perspectives.

While previous evidence has identified longer average diagnostic delays for CD than for UC,^5,6^ there were no notable differences in the present study data between those participants with CD and UC, with similar barriers to diagnosis identified regardless of condition. It may be that any differences based on diagnosis were difficult to unpick in a relatively small sample, and this could be a useful area to explore in future research in a larger participant sample. Beyond this, further research is needed to understand how different lengths of delay for both UC and CD may be associated with patient outcomes and, therefore, what length of delay is clinically significant. Research looking to develop interventions for use in primary care to improve identification and time to diagnosis could also positively affect patient outcomes.

It is important to note that the data in this study were collected prior to the COVID-19 pandemic. The post-COVID period has seen an increase in time to diagnosis for other gastrointestinal conditions (for example, colorectal cancer^18^) owing to service disruption and increased waiting times, and it seems likely that delays in diagnosis of IBD will have also increased, though further research on this is needed. The COVID-19 pandemic also brought about changes in primary care consulting, with remote consulting becoming increasingly common.^19^ Perspectives on remote consulting are mixed, but both GPs and patients have highlighted challenges in carrying out remote examinations,^20^ and GPs have highlighted the benefits of visualising signs and symptoms in person.^19^ Future research could usefully explore the impacts these changes may have had on diagnosis of IBD from the perspectives of patients and healthcare professionals.

To conclude, exploring individuals’ perceptions towards delayed diagnosis of their IBD led to the identification of several barriers to timely diagnosis at the patient, primary care, and systemic levels, which a number of patients felt had negatively impacted the progression of their condition and its management. These findings can support patients and GPs in their conversations about symptoms that may indicate IBD, and potentially contribute to reduce the delay in patients receiving a diagnosis.

## Data

In line with the Standard Operating Procedures in place at Keele School of Medicine, where this study was conducted, data are archived at a dedicated location within Keele University’s network. A request to access archived data can be made by completion of a Data Transfer Request form, which can be accessed by contacting: Primary Care Centre Versus Arthritis, School of Medicine, Keele University, Staffordshire ST5 5BG, UK; Tel: +44(0)1782733905.
